# Learning a confidence score and the latent space of a new supervised autoencoder for diagnosis and prognosis in clinical metabolomic studies

**DOI:** 10.1186/s12859-022-04900-x

**Published:** 2022-09-01

**Authors:** David Chardin, Cyprien Gille, Thierry Pourcher, Olivier Humbert, Michel Barlaud

**Affiliations:** 1 Transporters in Imaging and Radiotherapy in Oncology (TIRO), Direction de la Recherche Fondamentale (DRF), Institut des sciences du vivant Fréderic Joliot, Commissariat à l’Energie Atomique et aux énergies alternatives (CEA), Université Côte d’Azur (UCA), Nice, France; 2 Laboratoire d’Informatique, Signaux et Systèmes de Sophia Antipolis (I3S), Centre de Recherche Scientifique (CNRS), Université Côte d’Azur (UCA), Sophia Antipolis, France; 3 Centre Antoine Lacassagne, Université Côte d’Azur (UCA), Nice, France

## Abstract

**Background:**

Presently, there is a wide variety of classification methods and deep neural network approaches in bioinformatics. Deep neural networks have proven their effectiveness for classification tasks, and have outperformed classical methods, but they suffer from a lack of interpretability. Therefore, these innovative methods are not appropriate for decision support systems in healthcare. Indeed, to allow clinicians to make informed and well thought out decisions, the algorithm should provide the main pieces of information used to compute the predicted diagnosis and/or prognosis, as well as a confidence score for this prediction.

**Methods:**

Herein, we used a new supervised autoencoder (SAE) approach for classification of clinical metabolomic data. This new method has the advantage of providing a confidence score for each prediction thanks to a softmax classifier and a meaningful latent space visualization and to include a new efficient feature selection method, with a structured constraint, which allows for biologically interpretable results.

**Results:**

Experimental results on three metabolomics datasets of clinical samples illustrate the effectiveness of our SAE and its confidence score. The supervised autoencoder provides an accurate localization of the patients in the latent space, and an efficient confidence score. Experiments show that the SAE outperforms classical methods (PLS-DA, Random Forests, SVM, and neural networks (NN)). Furthermore, the metabolites selected by the SAE were found to be biologically relevant.

**Conclusion:**

In this paper, we describe a new efficient SAE method to support diagnostic or prognostic evaluation based on metabolomics analyses.

## Background

Deep neural networks have proven their effectiveness in bioinformatics for classification and feature selection [1–5]. They have also been recently used in metabolomic studies [6–10]. Classical stacked autoencoders [11] were used recently in metabolomic studies [12].

Autoencoders were introduced within the field of neural networks decades ago, their most efficient application at the time being dimensionality reduction [13, 14]. Autoencoders have also been used for denoising different types of data [11] to extract relevant features. One of the main advantages of the autoencoder is the projection of the data in the low dimensional latent space.

These autoencoder models include variational autoencoders (VAE) [15]. VAE networks encourage the latent space to fit a prior distribution, like a Gaussian. This can alter the accuracy of the model. In order to cope with this issue, some recent papers have proposed latent spaces with more complex distributions (e.g. mixtures of Gaussians [16]) on the latent vectors, but they are non-adaptive and unfortunately may not match the specific data distribution.

In this work, we relaxed the parametric distribution assumption on the latent space to learn a non-parametric data distribution of clusters [17]. Our network encourages the latent space to fit a distribution learned with the clustering labels rather than a parametric prior.

Recent untargeted metabolomic methods using liquid chromatography coupled with high resolution mass spectrometry (LC-MS/MS) allow for fast and high-resolution detection of massive amounts of metabolites. Metabolomics is a very promising omics field for fundamental research in biology as well as for clinical research applications. Indeed, metabolomics can be used to reveal new biomarkers of physiological or pathological states [18–21], and could be particularly useful for personalized medicine [22, 23].

In this study, we described a new SAE method using structured constraints and compare its performances to classical machine learning and Neural Network methods, when applied to three clinical metabolomic databases.

## Methods

### A New supervised Autoencoder (SAE) framework

Projecting the samples in the lower dimension latent space is crucial to separate them accurately. Herein we propose to use a neural network autoencoder framework.

Let us recall that the encoder part of the autoencoder maps feature-points from a high dimensional space to a low dimensional latent space, and that the decoder maps feature points from that low dimensional space to a high dimensional space.

**Fig. 1:**
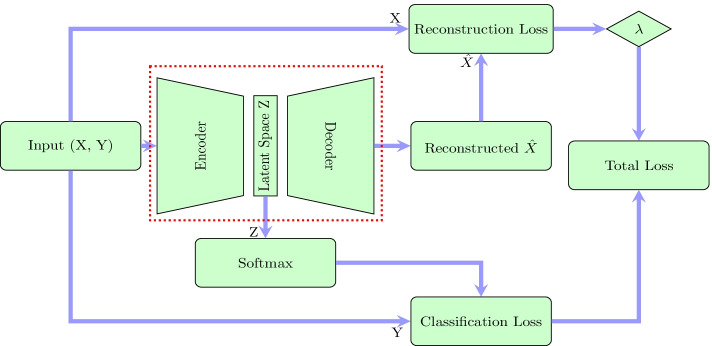
Supervised autoencoder framework

Figure 1 depicts the main constitutive blocks of our proposed approach. We have added to our SAE a “soft max” block to compute the classification loss.

Let *X* be the dataset, as an $m \times d$ data matrix made of *m* line samples $x_1,\dots ,x_m$. Let $y_{i}=j, j \in [1...k]$ be the label, indicating that the sample $x_i$ belongs to the *j*-th cluster. Let *Z*, be the latent space, $\widehat{X}$ the reconstructed data (Fig. 1) and *W* the weights of the neural network.

The goal is to compute the weights W minimizing the total loss, which depends on both the classification loss and the reconstruction loss. Thus, we propose to minimize the following criterion to compute the weights *W* of the autoencoder (see [17] for details).

1 $$\begin{aligned} Loss(W) = \phi ( Z,Y)+ \lambda \psi (\widehat{X} -X) \text { s.t. } \Vert W\Vert _1^1 \le \eta . \end{aligned}$$

Where $\phi ( Z,Y)$ is the classification loss in the latent space and $\psi (\widehat{X}-X)$ is the reconstruction loss.

The parameter $\lambda$ controls the weight of the reconstruction loss in the criterion. We used the Cross Entropy Loss for the classification loss function $\phi$. We used the robust Smooth $\ell _1$ (Huber) Loss [24] as the reconstruction loss function $\psi$, as it is more robust to outliers than the classical Mean Squared Error (MSE) loss. The dimension of the latent space is defined by the number of clusters.

### Structured constraints, sparsity and feature selection

The basic idea for feature selection is to use a sparse regularizer that forces some coefficients to be zero. To achieve feature selection, classically, the Least Absolute Shrinkage and Selection Operator (LASSO) formulation [25–29] is used to add an $\ell _1$ penalty term to the classification loss. However the LASSO is computationally expensive [26, 27]. Thus, we used a feature selection method by optimizing a criterion under constraints [30].

Let us recall that the classical $\ell _{2}$ norm constraint does not induce any sparsity. Moreover the “group Lasso $\ell _{2,1}$ constraint” induces small sparsity [31] and the $\ell _{1}$ constraint induces unstructured sparsity [32, 33]. Thus we used $\ell _{1,1}$ constrained regularization penalty $\Vert W\Vert _1^1 \le \eta$ for feature selection [17].

#### Algorithm

We compute the $\ell _{1,1}$ constraint with the following algorithm: we first compute the radius $t_i$ and then project the rows using the $\ell _1$ adaptive constraint $t_i$.

Following the work developed by [34], which proposed a double descent algorithm, we replaced the thresholding by our $\ell _{1,1}$ projection and devised a new double descent algorithm (See Barlaud and Guyard [35]) as follows :
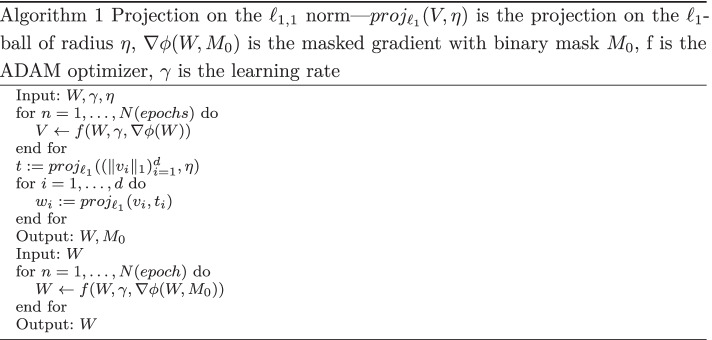


### Implementation

#### Pytorch implementation of our supervised autoencoder

We implemented our sparse supervised autoencoder model in the Pytorch framework. The losses are averaged across observations for each batch. We chose the ADAM optimizer [36], as the standard optimizer in PyTorch. We used the Netbio SAE, a linear fully connected network (LFC), which has an input layer of d neurons, 1 hidden layer of 96 neurons followed by a ReLU activation function, and a latent layer of dimension 2 (the number of classes). The parameter $\eta$ is determined by the maximum accuracy after cross-validation.

We compared the Netbio SAE with a classical linear fully connected Neural Network (NN) with the same structure.

We used the captum package [37] to compute the feature weights of the SAE.

We provide comparisons with a PLS-DA using 4 components, with Random Forests using 400 estimators and a maximum depth of 3 (using the Gini importance (GI) for feature ranking), and with a support vector classifier (SVM) with a linear kernel. For the SVM, we perform a cross-validation grid search to find the best regularization parameter C.

We provide the statistical evaluation (Accuracy, AUC, and F1 score) using a 4-fold cross validation process: the dataset is randomly divided into four parts, and trained on three of the four splits. The metrics are computed on the remaining test split, which wasn’t used during training. We then repeat this process three more times, leaving a different split as the test set each time. The final metrics given in this paper are averages over the four cross-validation steps, over three different random seeds (12 different testing/training splits in total).

We compare the performances of the different methods using the F1 Score. The F1 Score is the weighted average of Precision and Recall. Therefore, this score takes both false positives and false negatives into account. The F1 score is more relevant than accuracy, especially for unbalanced datasets.

The computation of the statistical metrics, the classifiers, the cross-validation function and the grid search were all provided by the scikit-learn machine learning python package. The python code is available on github: https://github.com/CyprienGille/Supervised-Autoencoder.

#### Diagnosis with confidence score

One of the main advantages of an autoencoder is the projection of the data in the latent space, which can easily be visualized if the latent space is of dimension 2.^1^ Thanks to this, we propose a clinical diagnosis simulation: having trained a network on a database of patients, we can predict a diagnosis with a confidence score for new patients. To perform this simulation, we removed a patient from each of the k classes from the databases. We then trained the SAE on (n-k) patients and we fed the k “test” patients through the best net. We thus obtained a visualization of the projections of these new “test” patients in the latent space as well as their classification with a confidence score (see Figs. 4, 10 and 7).

The clinician then has an accurate and reliable system to help with the diagnosis. Indeed, in addition to obtaining the confidence score for the diagnosis, the clinician can see where the patient is located among the others in the database and have a critical evaluation of the prediction (the clinician can easily see if a patient stands out).

### Evaluation on 3 clinical metabolomics databases

The SAE was tested on three different metabolomic datasets : the “LUNG” , “BREAST”, and “BRAIN” datasets.

The LUNG dataset was published by Mathe et al. [38] and is available at MetaboLights (study identifer MTBLS28). It includes metabolomics data concerning urine samples from 469 Non-Small Cell Lung Cancer (NSCLC) patients prior to treatment and 536 controls collected from 1998 to 2007 in seven hospitals and in the Department of Motor Vehicles (DMV) from the greater Baltimore, Maryland area. Urine samples were analyzed using an unbiased metabolomics LC-MS/MS approach. Mathe et al. used Random Forests to classify patients as lung cancer patients or controls [38]. The aim was to create a new screening test for lung cancer, based on metabolomics data from urine. Lung cancer is one of the most common cancers and it is well established that early diagnosis is crucial for treatment. An efficient screening method based on urinary metabolomics could be of great benefit.

The BREAST dataset was kindly provided by Dr. Jan Budczies and can be found in the supplementary material of Budczies et al. [39]. It includes metabolomics data concerning 271 breast tumor samples: 204 tumors with over-expression of estrogen receptors (ER) and 67 tumors without over-expression of ER. Metabolomics analysis was performed using Gas chromatography followed by time of flight mass spectrometry as described in [40].

The BRAIN dataset was obtained through a study performed in our lab$^{1*}$. It includes metabolomic data obtained on 88 frozen samples of glial tumors. The samples were retrospectively collected from two declared biobanks from the Central Pathology Laboratory of the Hospital of Nice and from the Center of Biological Resources of Montpellier (Plateforme CRB-CHUM). Consent or non-opposition was verified for every participant. Tumors were analyzed using Liquid Chromatography coupled to tandem Mass Spectrometry (LC-MS/MS) in an unbiased metabolomics approach. The details of the analysis are available in Additional file 1.

With this dataset, the goal was to create a model that accurately discriminated between mutated isocitrate dehydrogenase (IDH) and IDH wild-type glial tumors. The dataset includes (38 IDH wild-type tumors and 50 IDH-mutant tumors). This mutation is a key component of the World Health Organization classifcation of glial tumors [29]. The mutational status is usually assessed by IDH1 (R132H)-specifc (H09) immunohistochemistry. Yet this technique can lead to False-Negative results, which can only be identified by sequencing. Thus an accurate metabolomic based test, able to assess the IDH mutational status, could be a promising additional diagnostic tool.

The characteristics of the three metabolomic datasets are presented in Table 1. We chose to study these databases for their diversity both in terms of the number of features and number of patients, to test the robustness of our method on different types of databases.

The LUNG dataset includes a very large number of patients (1,005), with an equivalently large number of features (2,944), and 2 classes. The BREAST dataset includes a midsize number of patients (271), with a small number of features (161), and 2 classes. The BRAIN dataset includes a limited number of patients (88), with a much higher number of features (7,022), and 2 classes.

**Table 1:** Overview of the datasets

Dataset	No. of samples	No. of features	Sample type
LUNG	1005	2944	Urine
BREAST	271	161	Tumor tissue
BRAIN	88	7022	Glial tumor tissue

## Results

### LUNG dataset

#### Statistical performances

As shown in Table 2 our SAE outperformed PLS-DA, Random Forests, SVM and NN by 4.58, 9.58, 9.63 and $2.74\%$ respectively for the F1 score. Note that we checked that increasing the number of trees for Random forests from 100 to 400 resulted in a small improvement in accuracy of only $1 \%$ while the computational cost increased by a factor of 3. The performances of the SAE were a little better when using an $\ell _1$ loss than when using an $\ell _2$ loss.

**Table 2:** **LUNG** dataset: Accuracy using 3 seeds and 4-fold cross validation: comparison with PLS-DA, Random Forest, SVM and NN

Lung	SAE $\ell _1$	SAE $\ell _2$	PLS-DA	RF	SVM	NN
Accuracy $\%$	81.22	80.46	76.56	72.47	76.26	78.27
AUC	80.98	80.29	76.85	74.46	78.37	77.94
F1 score	80.74	80.29	76.16	71.16	71.11	78.00

#### Feature selection using the $\ell _{1,1}$ structured constraint

**Fig. 2:**
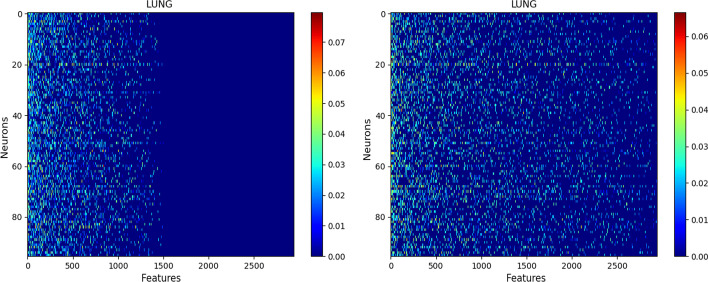
**LUNG** SAE Netbio Matrix: features versus hidden layer:Left with $\ell _{1,1}$ constraint,Right with $\ell _{1}$ constraint

Figure 2 shows the matrix ($d \times n$) of the network connections between the input layer (*d* feature neurons) and the hidden layer (*n* neurons).

It shows the benefit of using the $\ell _{1,1}$ constraint: The $\ell _{1,1}$ constraint selects features while the constraint $\ell _{1}$ selects only weights of features. All the following results are given with the $\ell _{1,1}$ constraint.

**Table 3:** Top 5 features on the **LUNG** dataset

SAE	PLS-DA	Random Forest	SVM	NN
MZ 264.12	MZ 264.12	MZ 264.12	MZ 170.06	MZ 264.12
MZ 308.09	MZ 126.90	MZ 441.16	MZ 126.90	MZ 126.90
MZ 126.90	MZ 613.35	MZ 584.26	MZ 264.12	MZ 308.09
MZ 232.03	MZ 170.06	MZ 486.25	MZ 94.06	MZ 613.35
MZ 332.09	MZ 243.10	MZ 204.13	MZ 110.99	MZ 332.09

From left to right: SAE, PLS-DA, Random Forest, SVM and NN

As shown in Table 3, all methods selected metabolite “MZ 264.121”, which most likely corresponds to creatine riboside (expected m/z value in the positive mode: 264.1190). Note that the SVM selected metabolite “MZ 264.121” at rank 3. Metabolite “MZ 308.098”, which most likely corresponds to N-acetylneuraminic acid, was only selected by the SAE and the NN at rank 2 and 3, respectively. These metabolites were described by Mathé et al. [38] as the most important metabolites to discriminate between lung cancer patients and healthy individuals. Note that the author of RF proposes two measures for feature ranking, the variable importance (VI) and Gini importance (GI): a recent study showed that if predictors are categorical, or real with multimodal Gaussian distributions, both measures are biased [41].

**Fig. 3:**
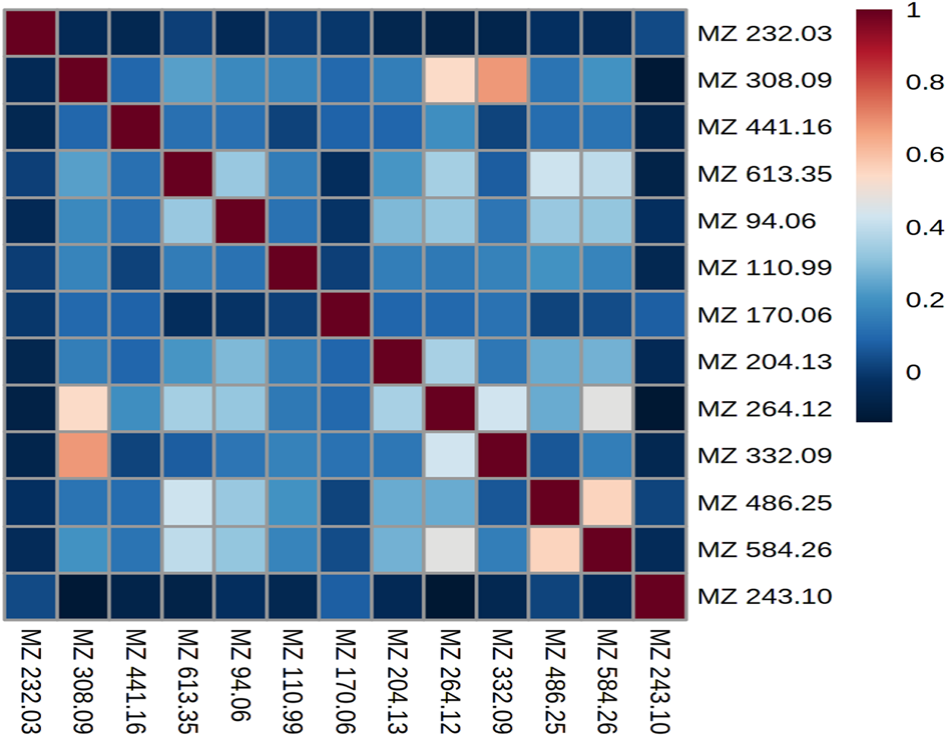
Correlation matrix of selected features in the **LUNG dataset**

As shown in Fig. 3, selected features were not significantly correlated. The highest correlation found was between MZ 308.09 and MZ 332.09, with a Pearson coefficient of 0.67. Both features correspond to adducts of N-acetylneuraminic acid (MZ 308.09 being the [M+H]+ adduct and MZ 332.09 the [M+Na]+ adduct).

#### Diagnosis in the latent space with a confidence score

**Fig. 4:**
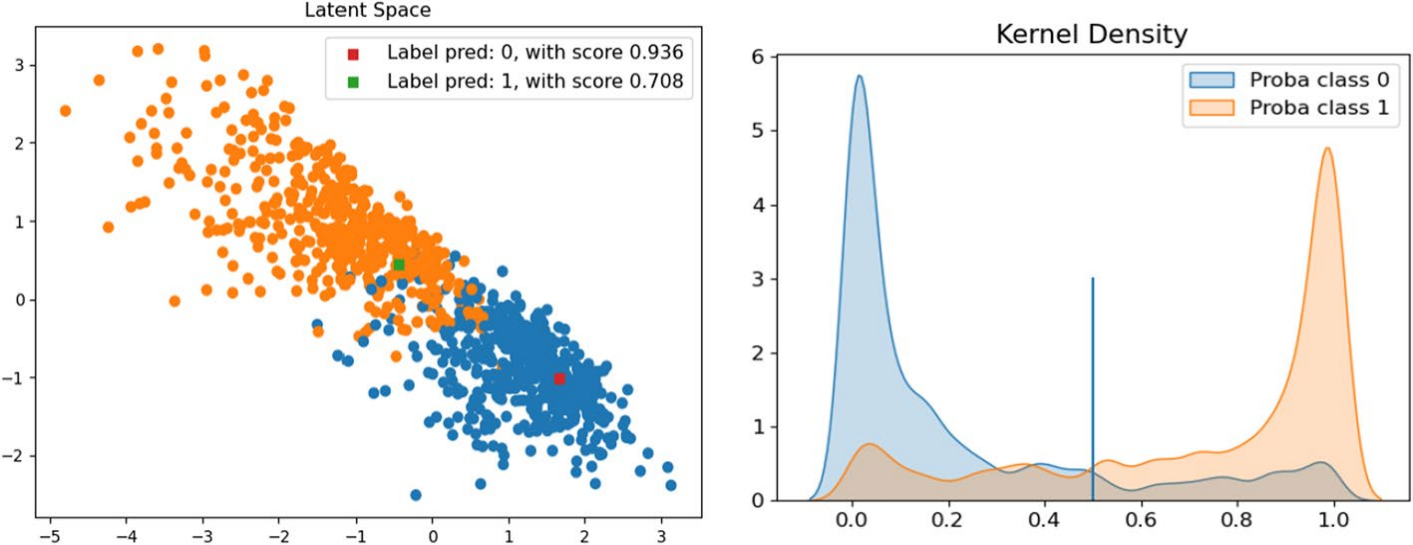
**LUNG** dataset. Right: Latent space, with test patients as squares. Left: Distribution using a Gaussian kernel

As shown in Fig. 4, the two classes are well separated in the latent space of the SAE. Furthermore, the red and green squares show the location of the two random “test” patients in the SAE’s latent space. The red patient is at the heart of the class distribution and the green patient is close to the edge of the other class. This is important for a clinician’s assessment of the result. Moreover, the distribution plot shows the nearly perfect separability of the distributions calculated with the SAE, which means most of the patients were diagnosed with a high degree of confidence. The patient represented by the red square was classified in class 0 with a confidence score of 0.94 and the patient represented by the green square was labeled class 1 with a confidence score of 0.70. Both predicted labels were correct.

### BREAST dataset

#### Statistical performances

**Table 4:** **BREAST** dataset: Accuracy using 3 seeds and 4-fold cross validation: comparison with PLS-DA, Random Forest, Logistic Regression, SVM and NN

Breast	SAE $\ell _1$	SAE $\ell _2$	PLS-DA	RF	SVM	NN
Accuracy $\%$	90.15	89.05	86.58	80.23	83.20	89.04
AUC $\%$	84.88	81.62	83.07	88.02	77.64	80.34
F1 Score	85.17	83.66	76.01	71.07	76.06	82.94

As shown in Table 4 our SAE outperformed PLS-DA, Random Forests, SVM and NN by 9.16, 14.1, 9.11 and $2.23\%$ respectively for the F1 score. The performances of the SAE were a little better when using an $\ell _1$ loss than when using an $\ell _2$ loss.

#### Feature selection using the $\ell _{1,1}$ structured constraint

**Fig. 5:**
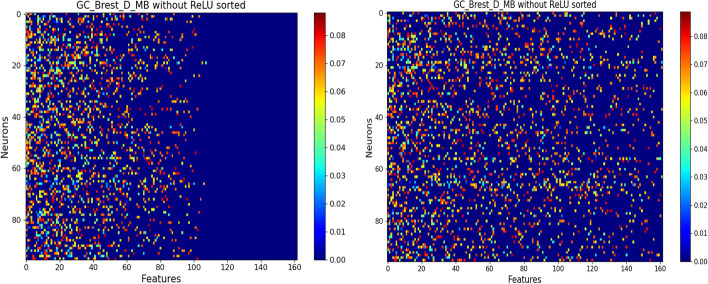
**BREAST** SAE Netbio Matrix: features versus hidden layer:Left with $\ell _{1,1}$ constraint,Right with $\ell _{1}$ constraint

Figure 5 shows the matrix ( $d \times n$) of the network connections between the input layer (d feature-neurons) and the hidden layer (n neurons). It shows the benefit of using the $\ell _{1,1}$ constraint: The $\ell _{1,1}$ constraint selects features, while the constraint $\ell _{1}$ selects only weights of features.

**Table 5:** Top 5 features on the **BREAST** dataset. From left to right: SAE, PLS-DA, Random Forest, SVM and NN

SAE	PLS-DA	Random Forest	SVM	NN
Beta-alanine	Beta-alanine	Beta-alanine	3-Phosphoglycerate	Beta-alanine
Xanthine	Xanthine	Xanthine	Beta-alanine	Xanthine
Uracil	Nicotinamide	glutamic acid	Uracil	2-hydroxyglutaric
Glutamic acid	Isothreonic acid	idonic acid NIST	Taurine	Uracil
2-Hydroxyglutaric acid	Creatinine	Uracil	2-Ketoadipic acid	Glutamic acid

As shown in Table 5, the SAE and the NN selected the same top five metabolites (beta-alanine, xanthine, uracil, glutamic acid). These metabolites have already been shown to have significantly different concentrations in ER− breast tumors compared to ER+ breast tumors in the original paper by Budczies et al. [39]. Increased concentrations of glutamic acid and 2-hydroxyglutaric acid indicate higher glutaminolysis, a key feature of metabolic changes in cancer cells. As shown in Budczies et al. [39], increased concentrations of uracil, xanthine and beta-alanine levels are related to higher hexokinase 3, xanthine dehydrogenase and 4-aminobutyrate aminotransferase expressions, respectively.

**Fig. 6:**
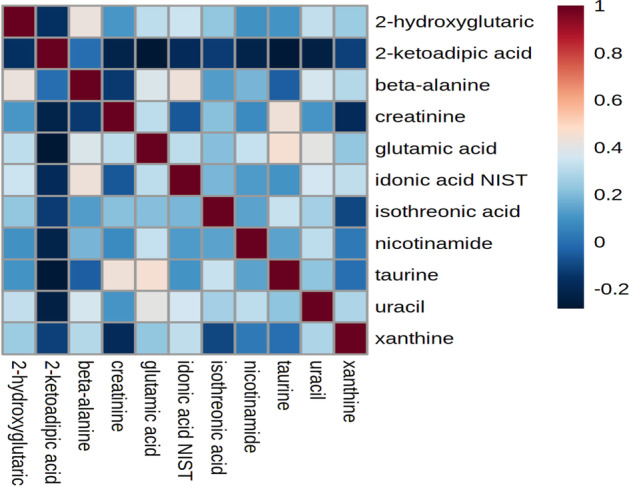
Correlation matrix of selected features in the **BREAST dataset**

As shown in Fig. 6, selected features were highly correlated.

#### Prognosis in the latent space with confidence score

**Fig. 7:**
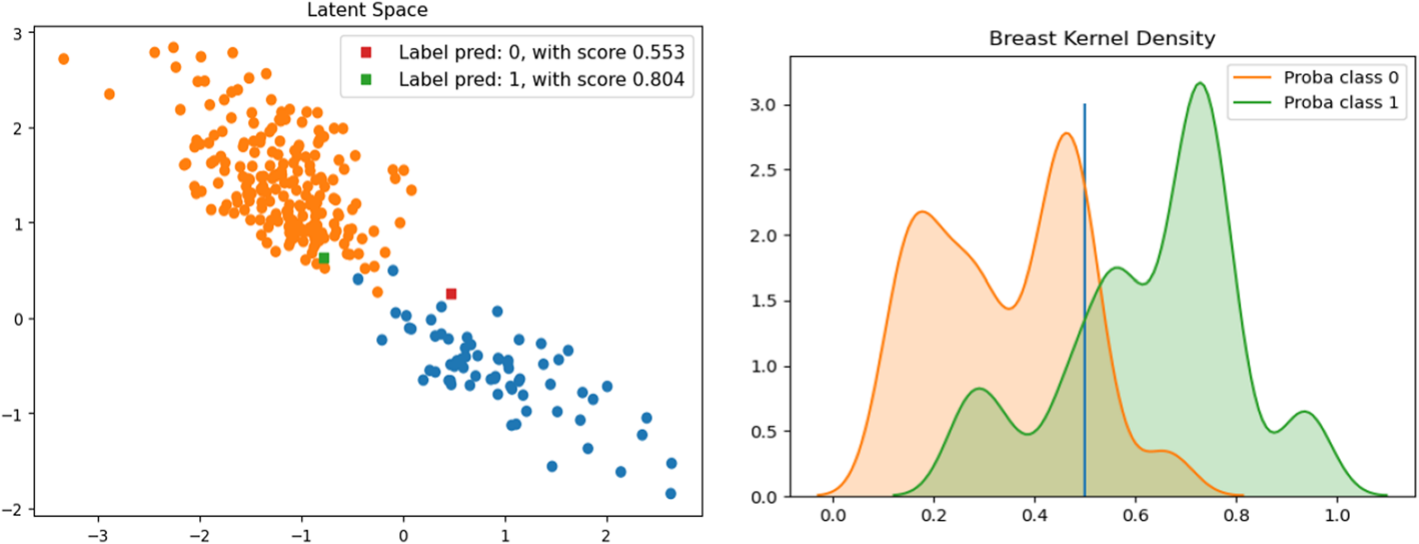
**BREAST** dataset. Left: latent space of the SAE. Right: Distribution using a Gaussian Kernel

Figure 7 (left), shows the accurate separation of the two classes in the latent space of the SAE. The red and green squares show the location of the two random “test” patients in the SAE’s latent space. The patient represented by the red square was classified in class 0 with a confidence score of 0.55 and the patient represented by the green square was labeled class 1 with a confidence score of 0.80. Both predictions are correct. Figure 7 (right) shows the separability of the distributions calculated with the SAE.

### BRAIN dataset

#### Statistical performances

**Table 6:** **BRAIN** dataset Accuracy using 3 seeds and 4-fold cross validation: comparison with PLS-DA, Random Forest , SVM and NN

Brain	SAE $\ell _1$	SAE $\ell _2$	PLS-DA	RF	SVM	NN
Accuracy $\%$	92.80	88.63	84.84	86.73	87.12	75.75
AUC $\%$	93.29	88.64	85.37	89.5	87.52	74.85
F1 score	92.66	88.40	83.88	88.05	86.51	74.19

Table 6 shows that, despite the small number of patients, the supervised autoencoder outperformed PLS-DA, Random Forest, SVM and NN by 8.78, 4.61, 6.15 and $18.47 \%$ respectively for the F1 score. For this base with few patients the performance of NNs collapses as reported in the literature. As for the other databases, the performances of the SAE were a little better when using an $\ell _1$ loss than when using an $\ell _2$ loss.

#### Feature selection using the $\ell _{1,1}$ structured constraint

**Fig. 8:**
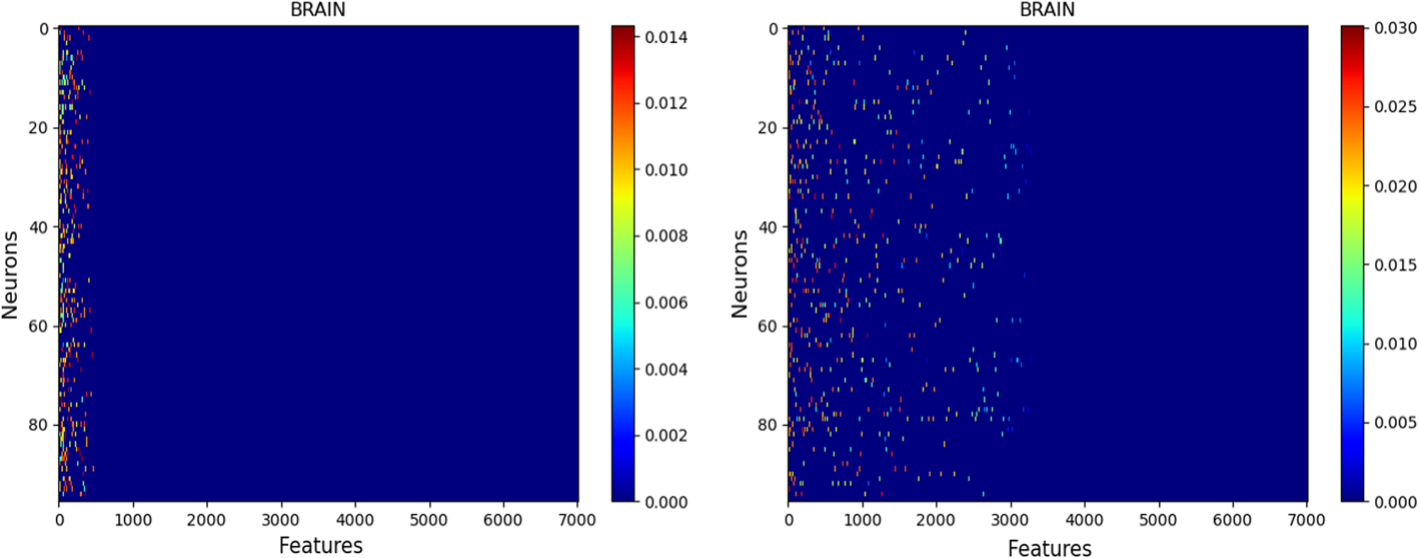
**BRAIN** SAE Netbio Matrix: features versus hidden layer: Left with $\ell _{1,1}$ constraint, Right with $\ell _{1}$ constraint

Figure 8 shows the matrix ( $d \times n$) of the network connections between the input layer (d feature-neurons) and the hidden layer (n neurons). It shows the benefit of using the $\ell _{1,1}$ constraint: The $\ell _{1,1}$ constraint selects features, while the constraint $\ell _{1}$ selects only weights of features.

**Table 7:** **BRAIN** dataset with 7,022 features : Top 5 features selected by the SAE, PLS-DA, Random Forests, SVM and NN

SAE	PLS-DA	RF	SVM	NN
NEG MZ147.028	POS MZ131.034	NEG MZ148.031	POS MZ132.523	NEG MZ148.031
POS MZ132.037	POS MZ132.523	NEG MZ215.016	NEG MZ147.028	NEG MZ147.028
POS MZ171.026	POS MZ132.037	POS MZ132.037	POS MZ131.034	POS MZ132.037
POS MZ132.037	NEG MZ147.028	POS MZ85.029	POS MZ132.037	POS MZ85.029
POS MZ149.044	POS MZ171.026	POS MZ132.523	POS MZ171.026	POS MZ173.030

**Fig. 9:**
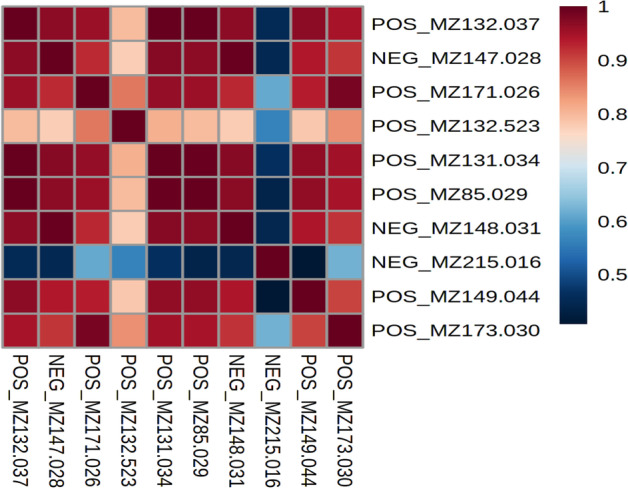
Correlation matrix of selected features in the **BRAIN dataset**

As expected, the top features selected by each method (shown in Table 7) correspond mainly to different isotopes and adducts of 2-hydroxyglutarate (marked in bold). The features selected using the SAE were all different adducts of this specific product of IDH-mutated cells. Indeed, POS_MZ132.03 and POS_MZ131.03 correspond to the [M+H-H2O]+ adduct of 2-hydroxyglutarate with one 13C isotope for the first ion. POS_MZ171.02 is the [M+Na]+ adduct, NEG_MZ147.02 is the [M-H]- and POS_MZ86.03 is the [M+Na+H]2+ adduct. NEG_MZ148.03 is the [M-H]- adduct of 2-hydroxyglutarate with one 13C isotope. POS_MZ173.03 is the [M+Na]+ adduct with two 13C isotope. Finally, POS_MZ149.04 is the [M+H]+ adduct ion of 2-hydroxyglutarate. As expected, and shown in Fig. 9, these features, all corresponding to adducts of 2-hydroxyglutarate, were highly correlated.

#### Diagnosis in the latent space with confidence score

**Fig. 10:**
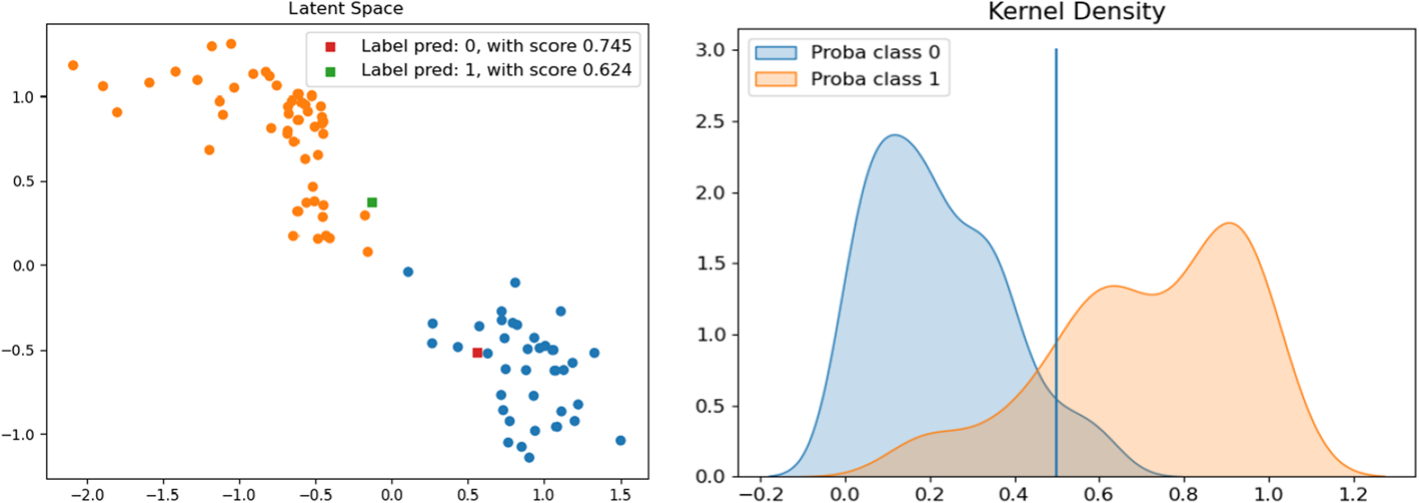
**BRAIN** dataset. Left: latent space of the SAE, Red and green squares are “test” patients. Right: Distribution using a Gaussian kernel

Figure 10 (left), shows the nearly perfect separation of the two classes in the latent space of the SAE. Furthermore, the red and green squares show the location of the two random “test” patients in the SAE’s latent space. The patient represented by the red square was classified in class 0 with a confidence score of 0.75 and the patient represented by the green square was labeled class 1 with a confidence score of 0.62. Both predictions were correct. Figure 10 (right) shows the peak separability of the distributions calculated with the SAE. It shows that most patients will have a good prediction with a high degree of confidence.

## Discussion

Thus, we have shown that our SAE outperformed classical machine learning methods and NN for classification of metabolomics data, while providing reliable confidence score for the predictions and performing relevant feature selection.

The real distributions of many datasets, including metabolomics datasets, are far more complex than multi-gaussian mixtures.Thus we chose to use a non-parametric supervised autoencoder (SAE) rather than a classical autoencoder that assumes a latent space modeling [42, 43] and force a multi-gaussian distribution upon the data.

Regardless of data size and feature space dimensions, the SAE outperforms all other methods (PLS-DA, Random Forests, SVM and NN). As expected, the NN also outperformed classical methods (PLS-DA, Random Forests and SVM), except on small databases. Indeed, NN are known to be less accurate when trained on small numbers of samples [44, 45]. Furthermore, as anticipated, the SAE’s performances were a little better when using the Huber loss than when using the MSE. This is most likely due to the fact that the Huber loss is more robust to outliers.

The SAE provides high-level distribution visualization of the samples in the latent space, as well as their classification confidence score. This is crucial for any diagnostic tool. Indeed, these two features enable clinicians to gauge how reliable each prediction is and if a sample corresponds to a potential outlier, for which predictions should be considered with particular care.

Metabolomics is a very promising approach, particularly adapted to routine clinical practice, because metabolomics analyses are fast and relatively inexpensive. However, human metabolomics are complex data, influenced by many external and internal factors. The high number of features included in metabolomics analyses require high performance statistical methods such as our SAE to be exploited. However, no statistical method can replace the critical reasoning of a researcher to make conclusions on the statistical results and to identify potential confounding factors. To make such conclusions, the statistical method needs to have some degree of interpretability.

Interestingly, the SAE combined with a structured projection provides efficient feature selection (Tables 3, 5 and 7). This feature selection step is crucial for interpretability. Better yet, we have verified that the selected features in the LUNG, BREAST and BRAIN datasets were known to be biologically relevant metabolites. Efficient feature selection adds interpretability to the model which is crucial for metabolomic studies in biological research or clinical trials.

We have observed that selected features can have a low to very high degree of correlation. In our case, the correlated features were isotopes and adducts of metabolites with high weights for the classification. Even though multivariate methods, such as the one we have used, account for correlation, correlated features do have an impact on feature selection and the performances of the trained models. When studying metabolomics one must adapt the level of filtering. Indeed, filtering removes isotopes and adducts but can also remove important features. This must be taken into consideration when using our SAE or any other classification method for metabolomics analyses.

## Conclusion

In this paper we have proposed a new and efficient classification method for metabolomics datasets, based on the representation of data on the latent space of a new supervised autoencoder (SAE). In clinical applications, our method provides a diagnosis score for each patient’s predicted class. Moreover, from a statistical point of view (Accuracy, AUC, F1 score) our SAE outperformed PLS-DA, Random Forest, SVM, and NN while selecting biologically relevant features.

## Data Availability

We implemented the code with python. Functions and scripts are freely available at https://github.com/CyprienGille/Supervised-Autoencoder. Furthermore the metabolomic databases are also available.

## Footnotes

1 If the latent space is of dimension $k>2$, we can project the latent space on a 2D plot using a PCA.
